# Factors associated with the depression status of Chinese parents who have lost their only child

**DOI:** 10.3389/fpubh.2022.931945

**Published:** 2022-08-24

**Authors:** Ya-Ping Ye, Jing-Na Wang, Qing-Chun Li, Cai-Ming Xu, Chao Rong

**Affiliations:** ^1^School of Humanities and Management, Zhejiang Chinese Medical University, Hangzhou, China; ^2^Hangzhou Center for Disease Control and Prevention, Hangzhou, China; ^3^School of Law, Zhejiang University City College, Hangzhou, China

**Keywords:** PLOCs, family planning policy, only child, depression, mental health

## Abstract

**Aim:**

This study aimed to assess the risk factors for depression among parents who have lost their only child (PLOCs).

**Methods:**

We used a cross-sectional survey to reveal the risk factors of depression among PLOCs. Multi-stage, stratified, cluster sampling was used to recruit the participants. The cluster sampling method was used to select PLOCs in Hangzhou, Zhejiang Province, and Wuhu, Anhui Province, while the stratified cluster sampling method was used in Anshun, Guizhou Province. A total of 651 PLOCs were recruited in this study. Participants completed the Social Support Rating Scale (SSRS) and the Geriatric Depression Scale-15 (GDS-15). Socio-demographics were also collected, including age, sex, monthly income, education level, marital status, self-reported health, and a number of diseases were collected as well. Chi-square tests and binary logistic regression were conducted to analyze the influence of these factors on PLOCs' mental status.

**Results:**

Two hundred and fifty-eight PLOCs (39.56%) reported depression. Compared to PLOCs living in Wuhu, those living in Hangzhou (OR = 3.374, CI = 2.337–4.870) had a higher risk of depression. Being single (OR = 1.449, CI = 1.019–2.061) and the presence/absence of grandchildren (OR = 0.430, CI = 0.274–0.676)were significantly associated with the depression status of PLOCs.

**Conclusion:**

The sampled Chinese PLOCs reported a high prevalence of depression that was influenced by their place of residence, marital status, and presence/absence of grandchildren. This may highlight the need for routine assessment and help of this group by the relevant stakeholders (including government, non-profit social organizations, and professional psychologists) with more attention paid to single and low-income PLOCs that have no grandchildren. It is imperative to build a comprehensive care system of “extended family—community—society—government” for this vulnerable group.

## Introduction

China's family planning policy has been in place for more than three decades, resulting in hundreds of millions of families having only one child. Scholars have estimated that there are ~170 million “only children” aged <44 years in current Chinese society (1). Although several decades of the “One-Child Policy” in China have played an important role in controlling population growth and stimulating economic growth, this policy has also led to the generation of a new vulnerable group—the Shidu family. When an only child dies early and the parents can no longer conceive or adopt another, this is referred to as a Shidu family (2). Parents who have lost their only child (PLOCs) are called Shidu parents in Chinese (3). In 2012, there were more than one million Shidu families in China (4), accounting for 2.5‰ of the total population. The total number of bereaved Shidu families is expected to reach 4.5 million by 2050 (5).

The concept of “raising children for old age” in Chinese culture has deep roots, with children taking on the responsibility for their parents in later life. In this context, PLOCs face great financial pressures (6) to cover the costs of their daily life and medical needs. Moreover, losing a child is one of life's greatest tragedies. Bereaved parents experience prolonged depression after the loss of a child (7). As a result, PLOCs are prone to loneliness, emptiness, and panic (8). People who experience such emotions for a long period of time are prone to depression. The existing research indicates that 80% of bereaved family members suffer from mental disorders or psychological trauma (9). Thus, there is a critical need for more attention and research on this topic (10). Depression is one of the most common mental disorders; it is characterized by low mood, physical discomfort, sleep disorders, and a cold and withdrawn personality (11), which may further lead to physical diseases (6). The event of losing an only child not only adversely affects the parents' mental health but also increases their medical burden and economic pressure, which eventually affects their physical health, thus resulting in a vicious circle.

Research suggests that the major risk factors for depression among PLOCs are low-education level, poor physical health status, low-income level, singlehood, and poor social support (12). Scholars have shown a keen interest in the association between social support and depression, revealing that social support is negatively associated with depression (13–15). Others have preferred to target the mental health of PLOCs through case-by-case interventions in small samples (16, 17). In the above-mentioned studies, cultural factors, such as “the third generation”, are largely ignored. Moreover, most of the available Chinese studies recruited participants from only one city in China. The samples of those studies may not sufficiently represent the situation in China as a whole. Therefore, a national investigation with more participants, incorporating more factors, is needed. A clear understanding of the pathways that lead to the onset of depression is critically a first step on the road to developing effective preventive measures. Therefore, the aim of the present study was to identify the risk factors for depression among PLOCs using a national sample. It was hoped that these findings would provide possible targets for medical and social workers to better support and care for this special group.

## Methods

### Sampling

Due to the large differences in economic development between different regions (18), China has been divided into three economic belts: eastern, central, and western belts. The present study took one city from each belt as a representative sample city. Multi-stage, stratified, cluster sampling was based on the economic belts, and representative cities in the eastern, central, and western belts, namely, Hangzhou, Wuhu, and Anshun, respectively, were chosen for sampling. In China, the urban administrative division is roughly divided into three levels: city, district, and community. Considering the representativeness and feasibility of the survey in each city, different sampling methods were applied. Stratified random sampling was used in Hangzhou and Anshun due to the huge development gap between different districts in these cities, while cluster sampling was used in Wuhu. In total, 651 PLOCs were recruited and analyzed.

Hangzhou was chosen as one of the sample cities for convenience. Data were collected from July to August 2017 *via* the stratified random sampling method. The sampling process involved a systematic two-step approach: (i) based on the Gross Domestic Product (GDP), the districts of Hangzhou were divided into three levels (high, medium, low), and two districts at each level, for a total of six districts, were chosen; (ii) based on the data provided by the District Health and Family Planning Bureau, two communities with the largest numbers of Shidu families were selected for sampling.

The cluster sampling method was used to select participants from Wuhu between April and July 2018. A systematic three-step sampling approach was adopted: (i) based on the administrative divisions, Wuhu city was divided into four districts: Jinghu, Jiujiang, Yijiang, and Sanshan; (ii) Jinghu district was selected from Wuhu because there are more PLOCs in this city; (iii) all communities in the Jinghu district were selected. All PLOCs in these communities were surveyed.

Finally, for the city of Anshun, based on the GDP, the districts of Anshun were divided into two levels (high, low) and one district was selected from each level. The stratum sampling method was used in the two selected districts, and on-site investigations were conducted in the communities with more PLOCs.

The inclusion criteria for this study were: (i) age 45 years and above, given that most PLOCs do not have another baby when they are over age 44 years; and (ii) normal cognitive function. The exclusion criteria were: (i) relocated elsewhere; (ii) parents who refused to accept the government's or others' condolences or investigations.

Trained professors, undergraduates, and local family planning officials worked as the interviewers and helped the participants to complete the questionnaires. The questionnaires contained three parts: sociodemographic characteristics, the Social Support Rating Scale (SSRS), and the Geriatric Depression Scale (GDS-15).

### Measurement

#### Dependent variable

The GDS-15 was used to measure the depression status of the respondents in the week preceding questionnaire completion. This scale measures participants' levels of irritability, low mood, reduced activity, pain, withdrawal and other thoughts, and their negative evaluations of the past, present, and future. The Cronbach's α coefficient of GDS-15 was 0.793, and the item-total correlation was 0.196–0.534 (19). In conclusion, the GDS-15 shows acceptable reliability and validity and can be used in research of depression symptoms among Chinese.

The score of GDS-15 was regarded as the dependent variable in this study. The participants were required to answer “Yes” or “No” to each item, with “Yes” assigned one point and “No” assigned zero points. A higher cumulative score on the GDS-15 indicates more severe depression. Scores of 3 and below, between 4 and 8, and 8 and above are generally defined as non-depression, minor depression, and severe depression, respectively. In the current study, scores of 3 and below were defined as non-depression, while 4 and above were defined as depression.

#### Independent variables

The independent variables included sex, age, educational level, marital status, monthly income, having grandchildren, self-reported health status, and social support.

The SSRS was used to measure the participants' social support. It consists of three dimensions: subjective supports (item 1, 3–5), objective supports (item 2, 6–7), and availability (item 8–10). Higher scores indicate higher levels of social support. A total score of <20 indicates a low level of social support, 20–30 indicates a fair level of social support, while >30 indicates satisfactory social support (20). The SSRS was designed and widely used to assess the social support status of Chinese. The SSRS demonstrated adequate internal consistency (Cronbach's alpha = 0.859) and construct validity (*r* = 0.715).

Number of chronic diseases was measured by a multiple-choice question, “How many chronic diseases do you have?” (response options: 1 = have no chronic disease, 2 = have one chronic disease, 3 = have two chronic diseases, and 4 = have no <3 chronic diseases; have chronic disease was used as the reference value). Chronic disorders or diseases were listed for reference, and included the following: hypertension, diabetes, malignant tumor, hyperlipidaemia, cerebral infarction (stroke), coronary heart disease, chronic liver disease, cerebrovascular disease, senile dementia, gout, asthma, tuberculosis, arthritis, gynecological disease, haematopathy, chronic low back pain, osteoporosis, and cataract.

#### Ethical approval

The Medical Ethics Committee of Zhejiang Chinese Medical University granted approval for this study. All participants were clearly informed of the purposes of this study and signed the consent form. All participants were assured of their right to refuse to participate or to withdraw from this study at any time. Confidentiality and anonymity of the participants were also assured.

#### Statistical analysis

SPSS version 21 software was used to analyze the data. The participants' sociodemographic data were described in terms of frequency and percentage. Chi-square tests and binary logistic regression analysis were performed to explore the associations between the independent variables and depression.

Before the binary regression analysis, chi-square tests were performed to control for confounding factors. Variables with *P* < 0.05 were included in the binary logistic regression analysis to explore the associations between these risk factors and depression status. In the results of binary logistic regression analysis, variables with *P* < 0.05 were considered risk factors.

## Results

### Characteristics of the participants

In total, 651 PLOCs participated in this study. The sociodemographic characteristics of the participants are shown in Table 1. Most participants were 60 years old and above (464; 71.3%). More than half of the participants (352; 54.1%) were female. A large proportion of participants only had a junior high school degree or below (467; 71.7%). The majority of participants had grandchildren (79.9%). Overall, 71.9% of the participants had at least one chronic disease, while only 62.4% had a good level of social support. More than one-third of the participants (39.6%) were depressed (Table 1).

**Table 1:** Participant characteristics and depression status.

**Variable**	**Frequency**	**Percentage (%)**
**Age**		
<60 years	187	28.7
≥60 years	464	71.3
**Gender**		
Male	299	45.9
Female	352	54.1
**City**		
Wuhu	306	47.0
Hangzhou	237	36.4
Anshun	108	16.6
**Monthly income (Yuan)**		
≤ 2,000	152	23.3
2,000–3,000	221	33.9
≥3,000	278	42.7
**Education level**		
Junior high school or below	467	71.7
Senior high school	125	19.2
College and above	59	9.1
**Marital status**		
Married	446	68.5
Unmarried	205	31.5
**Self-reported health status**		
Good	262	40.2
Fair	319	49.0
Poor	70	10.8
**Have grandchildren?**		
No	520	79.9
Yes	131	20.1
**Have one or more chronic diseases?**		
No	183	28.1
Yes	468	71.9
**Social support status**		
Poor	24	3.7
Fair	221	33.9
Good	406	62.4
**Depression status**		
No depression	393	60.4
Depression	258	39.6

### Chi-square tests and binary logistic regression analysis

Table 2 shows the results of the chi-square tests for the factors affecting the depression status of PLOCs. City (*P* < 0.001), income (*P* = 0.009), marital status (*P* = 0.031), and having grandchildren (*P* = 0.001) were significantly associated with the depression status of PLOCs.

**Table 2:** Chi-square test results.

**Independent variables**	**Depression**	**Non-depression**	**Depression Rate**	**χ^2^**	***P***
**Age**					
<60 years	84	103	44.9	3.067	0.080
≥60 year	174	290	37.5		
**Gender**					
Male	108	191	36.1	2.849	0.108
Female	150	202	42.6		
**City**					
Wuhu	94	212	30.7	44.824	<0.001
Hangzhou	134	103	56.5		
Anshun	30	78	27.8		
**Monthly income (Yuan)**					
≤ 2,000	52	100	34.2	9.314	0.009
2,000–3,000	77	144	34.8		
≥3,000	129	149	46.4		
**Education level**					
Junior high school or below	178	289	38.1	2.161	0.339
Senior high school	52	73	41.6		
College and above	28	31	47.5		
**Marital status**					
Married	164	282	36.8	4.842	0.031
Unmarried	94	111	45.9		
**Self-reported health status**					
Good	106	156	40.5	4.384	0.112
Fair	117	202	36.7		
Poor	35	35	50.0		
**Have grandchildren?**					
No	222	298	42.7	10.120	0.001
Yes	36	95	27.5		
**Have one or more chronic diseases?**					
No	73	110	39.9	0.007	0.929
Yes	185	283	39.5		
**Social support status**					
Poor	13	11	54.2	3.907	0.142
Fair	94	127	42.5		
Good	151	255	37.2		

The above statistically significant variables were then included in a binary logistic regression model to further test their independence. City (OR = 3.374, CI = 2.337–4.870, *P* < 0.001), marital status (OR = 1.449, CI = 1.019–2.061, *P* = 0.039), and having grandchildren (OR = 0.430, CI = 0.274–0.676, *P* < 0.001) were significantly associated with depression status (Table 3).

**Table 3:** Binary logistic regression analysis of factors influencing depression status among PLOCs.

**Independent variables**	**β**	**OR (95%CI)**	***P***
**City**			
Wuhu	Ref	Ref	Ref
Hangzhou	1.216	3.374 (2.337, 4.870)	<0.001
Anshun	0.112	1.119 (0.674, 1.857)	0.664
**Marital status**			
Married	Ref	Ref	Ref
Unmarried	0.371	1.449 (1.019, 2.061)	0.039
**Have grandchildren?**			
No	Ref	Ref	Ref
Yes	−0.843	0.430 (0.274, 0.676)	<0.001

## Discussion

The findings of the current study provide insight into the prevalence and risk factors of depression among PLOCs in China. Based on data from the China Family Panel Studies (CFPS) in 2018, the national rate of depression among middle-aged and elderly people aged 45 years and above is estimated to be 23.61% (21). The rate of depression among the PLOCs in our study (39.6%) was higher than this national estimate. Moreover, the current results revealed that the prevalence of depression among PLOCs in Hangzhou was higher than that among PLOCs in Wuhu. PLOCs having grandchildren were less likely to be depressed, while those living in Hangzhou and those who were unmarried were at higher risk of depression.

The results of this study indicated that the depression rate in Hangzhou was higher than that in Wuhu. This suggests that the rate of depression varies from eastern to central China. As noted previously, the level of economic development, education, employment, urbanization, and the living environment all significantly impact human health (22). The existing studies suggest that income inequality is one of the most important driving factors in health inequality (23). Most previous studies have reported a significant positive correlation between socioeconomic status and health (24, 25). However, there is no consensus on this among PLOCs from different regions in China due to the particularity of this group. Although the economic status and education level of residents in eastern China are generally better than those of residents in central China (26), our data demonstrated that eastern residents were more likely to experience depression. According to Maslow's hierarchy of needs theory, individuals only pursue higher-level needs after lower-level needs are satisfied. The great grief caused by losing a child has a primary effect on emotional needs, which is higher in the hierarchy than physical and safety needs (27). Therefore, PLOCs in eastern China may be at higher risk of experiencing complications from depression while those living in the central region may pay more attention to making a living.

Economic development has led to different levels of urbanization in different regions. The degree of urbanization in the eastern region is higher than that in the central region (28). In the process of urbanization, traditional neighborhood relations are also constantly changing (29). As a modern community becomes the product of urbanization, neighborhood relations are fairly similar compared to the traditional social unit (30, 31). Emotional support from neighbors is regarded as a positive factor in recovery from depression among PLOCs (32). Under this circumstance, PLOCs in Hangzhou may receive less emotional support from their neighbors than PLOCs in Wuhu, which may result in a poorer psychological state.

With respect to marital status, the current study indicated that the rate of depression among PLOCs without a spouse was higher than the rate among married people. Research spanning several decades has demonstrated that people benefit from marriage, in terms of both physical and mental health, as compared with non-married individuals (33, 34). Both spouses and children are critical components of one's social network (35). Following the loss of a child, the spouse becomes an individual's closest person in later life (36). The breakdown of the marriage undoubtedly adds to the misfortunes of PLOCs, and the emotional support they could receive will also be reduced, which can have a detrimental effect on their mental health. On the other hand, a failing marriage may in itself be a source of stress (37) and has been shown to be a trigger for depression (38).

The current study also revealed that the rate of depression among PLOCs in China was negatively correlated with having grandchildren. Compared to the participants who had no grandchildren, those with grandchildren were less likely to experience depression, which is similar to previous studies (39, 40). Offspring are regarded as a continuation of family affection. Grandchildren are a source of strong emotional support, especially for PLOCs. Moreover, raising grandchildren enriches one's life. The role of caregiving enhances a grandparent's sense of purpose in life (41). Over time, the pattern and norms of child-raising have changed. PLOCs must learn the goals of today's parents (42) and consider how to get along with their grandchildren, which may divert their attention from the pain of losing their only child. The transition into this role leads to new life goals for PLOCs, and caring for their grandchildren may help them to regain a meaningful life (43). Moreover, through interaction and communication with grandchildren and others, loneliness and boredom among the elderly can be relieved, if not completely eliminated (44).

## Limitations

The present study has two limitations that should be noted. First, since it was a cross-sectional study, no causal inferences can be drawn. Second, this study only analyzed PLOCs without a comparison with non-PLOCs; a comparative study is warranted in the future. Future studies are supposed to continue to explore the long-term impact of the loss of an only child.

## Conclusion

The sampled Chinese PLOCs reported a high prevalence of depression that was influenced by their place of residence, marital status, and presence/absence of grandchildren. This study focused on the mental health of PLOCs and revealed that not only personal factors but social factors also affect the mental health of PLOCs. Thus, apart from focusing on individuals, strong and professional social support and generous social welfare are critical to alleviating the psychological trauma experienced by PLOCs. First, the government is recommended to improve the national welfare system and reasonably take PLOCs into consideration to address their main living concerns. Second, non-profit social organizations should also play an important role, such as dispatching social workers and psychological volunteers to provide psychological counseling and other mental health services. Furthermore, encouraging community workers and extended family members to establish an accessible and more intimate psychological support network will be helpful to PLOCs. With these approaches, an “extended family—community—society—government” model, a comprehensive mental health care system can be designed for this vulnerable group.

## Data availability statement

The raw data supporting the conclusions of this article will be made available by the authors, without undue reservation.

## Ethics statement

Ethical review and approval was not required for the current study in accordance with the local legislation and institutional requirements. The Medical Ethics Committee of Zhejiang Chinese Medical University decided that a full review was not necessary, and decided to grant ethical approval waiver. The participants provided their written informed consent to participate in this study. All participants were assured of their right to refuse to participate or to withdraw from this study at any time. Confidentiality and anonymity of the participants were also assured.

## Author contributions

CR contributed to the conception of the study. J-NW contributed significantly to analysis and manuscript preparation. Y-PY performed the data analyses and wrote the manuscript. C-MX helped perform the analysis with constructive discussions. Q-CL checked the full text and made some revisions. All authors contributed to the article and approved the submitted version.

## Funding

This study was partially funded by the 2021 Zhejiang University Student Science and Technology Innovation Activity Plan (Xinmiao Talent Plan) (2021R410064), the special project on the cultivation of leading talents in social sciences in Zhejiang Province, China “Research on evaluation and promotion strategy of comprehensive service ability of traditional Chinese medicine's characteristic home-based combination of medical care and elderly care driven by Big Data” (21QNYC16ZD), and Social Science Youth Exploration Project of Zhejiang Chinese Medical University (2021JKSKTS003A).

## Conflict of interest

The authors declare that the research was conducted in the absence of any commercial or financial relationships that could be construed as a potential conflict of interest.

## Publisher's note

All claims expressed in this article are solely those of the authors and do not necessarily represent those of their affiliated organizations, or those of the publisher, the editors and the reviewers. Any product that may be evaluated in this article, or claim that may be made by its manufacturer, is not guaranteed or endorsed by the publisher.

## References

[1] Feng XT. The one-child problem in the post-one-child era. J Zhejiang Academic. (2020) 5:64–73. 10.16235/j.cnki.33-1005/c.2020.05.008

[2] Li X Zhu HB. A brief analysis of the way of spiritual support for families with “lost only child”. Manag Observ. (2014) 34:170–1. 10.3969/j.issn.1674-2877.2014.34.072

[3] Yin Q Shang Z Zhou N Wu L Liu G Yu X. An investigation of physical and mental health consequences among Chinese parents who lost their only child. BMC Psychiatry. (2018) 18:45. 10.1186/s12888-018-1621-2 29433470 PMC5810017

[4] Xiao L Sun M Wang QY Tang SY. The dilemma and the countermeasures faced by the families of losing their only child in China. Chin J Gerontology. (2016) 36:742–4. 10.3969/j.issn.1005-9202.2016.03.101

[5] Wang GZ. Research on the total population, age structure and developing trend of the lost only child women by computer simulation. Popul Econ. (2016) 37:1–11. 10.3969/j.issn.1000-4149.2016.05.001

[6] Xu CM Rong C Tan JG. Recent developments in the delivery of integrated medical and nursing care for elderly parents bereaved of their only child. Chin Genl Practice. (2018) 21:1932–7. 10.3969/j.issn.1007-9572.2018.16.006

[7] Ramstedt M Hope A. The Irish drinking habits of 2002-drinking and drinking-related harm in a European comparative perspective. J Subst Use. (2005) 10:273–83. 10.1080/14659890412331319443

[8] Wu RF Xiong ZW Zhu XT. Study on the status quo and countermeasures of mental endowment for the elderly who lost their only child in town—taking C City of J Province as an example. Reform Openning. (2016) 31:81–2. 10.16653/j.cnki.32-1034/f.2016.16.045

[9] Gu CC. Optimizing of the Role of Government in the Old-age Security of the Family Which Lost the Only Child. Chengdu: University of Electronic Science and Technology of China (2016).

[10] Li JS Ma WJ. Prevalence and influencing factors of depression symptom among middleaged and elderly people in China. Chin J Public Health. (2017) 33:177–81. 10.11847/zgggws2017-33-02-02

[11] Hu PZ. A Case Study of Depression in the Elderly Who Lost Their Only Child – A Case Study of L Elderly in Donghu District, Nanchang City. Nanchang: Jiangxi University of Finance & Economics (2017).

[12] Huang QD. A review of studies on mental depression among people who lost their only child. Yangtze Tribune. (2020) 4:68–73. 10.3969/j.issn.1005-3980.2020.04.011

[13] Zhao S Long MJ Diao YC Ma HF Liu MH Feng ZY. Culture-related grief beliefs and social support influence depressive symptoms of Shidu parents in rural China. Eur J Psychotraumatol. (2021) 12:1945748. 10.1080/20008198.2021.1945748 34367525 PMC8312613

[14] Wang E Hu H He Y Xu Y. Can social support matter? The relationship between social support and mental health among bereaved parents in an only-child society: Evidence from China. Health Soc Care Community. (2021) 29:476–86. 10.1111/hsc.13108 32701221

[15] Hu PZ. Interventional Study on the Case of Depression in Elderly People Who Lost Single Child-A Case Study of L Elder People in Donghu District of Nanchang. Jiangxi: University of Finance and Economics (2017).

[16] Fang SG. The psychological comfort for only-child-died-elderly under the case involvement of social work. J Dezhou Univ. (2017) 33:42–8. 10.3969/j.issn.1004-9444.2017.03.009

[17] Yang R Li YF. From despair to rebirth: action research of social work intervening for the elderly people who lost the only child. Social Cons. (2018) 5:32–41. Available online at: http://www.cqvip.com/qk/72016x/201801/674378689.html

[18] Li HN Ye MQ. An analysis of the economic development gap between the east, middle and west regions of China. Inquiry Into Econ Issues. (2006) 2:4–11. 10.3969/j.issn.1006-2912.2006.02.001

[19] Tang D. Application of short form geriatric depression scale (GDS-15) In Chinese Elderly. Chin J Clin Psychol. (2013) 21:402–5. 10.16128/j.cnki.1005-3611.2013.03.036

[20] Zhang W Wang A Guo Y Yao S Zhang J. Mediation role of self-efficacy between social support and depression of only-child-lost people. J Cent South Univ (Med Sci). (2017) 42:836–42. 10.11817/j.issn.1672-7347.2017.07.016 28845010

[21] Wu NW Yang F Xia J Ma TP Yu C Li XN. Analysis of the status of depression and the influencing factors in middle-aged and older adults in China. J Sichuan Univ (Med Sci). (2021) 52:767–71. 10.12182/20210960507 34622590 PMC10408879

[22] Li W Xiang Y Zhu TY. Analysis of the influence of social determinants on the health level of residents in the eastern, central and western regions. Modern Business. (2017) 1:250–2. 10.14097/j.cnki.5392/2017.01.127

[23] Doorslaer Ev Koolman X. Explaining the differences in income-related health inequalities across European countries. Health Econ. (2004) 13:609–28. 10.1002/hec.918 15259042

[24] Wang FQ. Socioeconomic status, lifestyle and health inequality. Chin J Sociol. (2012) 32:125–43. 10.15992/j.cnki.31-1123/c.2012.02.001

[25] Feinstein JS. The relationship between socioeconomic status and health: a review of the literature. Milbank Q. (1993) 71:279–322. 10.2307/3350401 8510603

[26] He YZ Wang HF Liu JW. An empirical analysis of regional economic development differentiation in chinese eastern, middle and western regions. J Nanchang Hangkong Univ:Social Sci. (2016) 18:48–54+112. 10.3969/j.issn.1009-1912.2016.03.008

[27] Liu TL Song XM Cheng FD Wang CS Zhang ZJ Chao R. The impact of resources from family and society on psychological well-being among elderly parents who lost their only child-based on perspective of Maslow's hierarchy of needs. Popul Dev. (2019) 25:87–93. Available online at: http://www.cqvip.com/qk/90419x/201904/7002575261.html

[28] Ma ZD Yu HX. An empirical analysis of the relationship between industrial agglomeration and urbanization-based on the perspective of the differences between east, middle and west my country. J Hebei Univ (Philos and Social Sci). (2016) 41:80–7. 10.3969/j.issn.1005-6378.2016.06.012

[29] Xue FF. Studies of the communication between neighborhoods in urban community. Archit J. (2004) 4:26–8. 10.3969/j.issn.0529-1399.2004.04.009

[30] Meng XY. Discussion on the relationship between neighbor relationship and community governance. Art Liter Masses. (2018) 14:241–2. 10.3969/j.issn.1007-5828.2018.14.175

[31] Hu XW Zhang LY Jin GM. Breaking and bridging: an empirical research on the neighborhood relationshil among urban residents in Nanjing. Urban Insight. (2020) 1:153–64. 10.3969/j.issn.1674-7178.2020.01.014

[32] Pan JH Hu CQ Hao RJ. An analysis on influence factors of parents who lost only child when going out from grief—based on the survey of 1084 parents who lost only child. Popul Dev. (2018) 24:72–83. Available online at: http://www.cqvip.com/qk/90419a/201805/676576912.html

[33] Carr D Springer KW. Advances in families and health research in the 21st century. JMF. (2010) 72:743–61. 10.1111/j.1741-3737.2010.00728.x

[34] Goldman N Korenman S Weinstein R. Marital status and health among the elderly. Soc Sci Med. (1995) 40:1717–30. 10.1016/0277-9536(94)00281-W 7660185

[35] Aartsen MJ. Tilburg Tv Smits CHM Knipscheer C. A longitudinal study of the impact of physical and cognitive decline on the personal network in old age. J Soc Pers Relat. (2004) 21:249–66. 10.1177/026540750404138634516176

[36] Antonucci TC Akiyama H Takahashi K. Attachment and close relationships across the life span. Attach Hum Dev. (2004) 6:353–70. 10.1080/1461673042000303136 15764124

[37] Stafford M Antonucci TC Zaninotto P. Joint trajectories of spousal social support and depressive symptoms in older age. J Aging Health. (2019) 31:760–82. 10.1177/0898264317747077 29254428 PMC6495403

[38] Mayer SE Lopez-Duran NL Sen S Abelson JL. Chronic stress, hair cortisol and depression: a prospective and longitudinal study of medical internship. Psychoneuroendocrinology. (2018) 92:57–65. 10.1016/j.psyneuen.2018.03.020 29627713 PMC5924646

[39] Tsai FJ Motamed S Rougemont A. The protective effect of taking care of grandchildren on elders' mental health? Associations between changing patterns of intergenerational exchanges and the reduction of elders' loneliness and depression between 1993 and 2007 in Taiwan. BMC Public Health. (2013) 13:567. 10.1186/1471-2458-13-567 23758624 PMC3689038

[40] Arpino B Bordone V Balbo N. Grandparenting, education and subjective well-being of older Europeans. Eur J Ageing. (2018) 15:251–63. 10.1007/s10433-018-0467-2 30310372 PMC6156724

[41] Giarrusso R Feng D Silverstein M Marenco A. Primary and secondary stressors of raising grandchildren: evidence from a national survey. J Ment Health Aging. (2000) 6:291–310. Available online at: https://psycnet.apa.org/record/2001-14827-002

[42] Strom RD Strom SK. Meeting the challenge of raising grandchildren. Int J Aging Hum Dev. (2000) 51:183–98. 10.2190/FR92-EGW2-VEVU-P8CR 11197732

[43] Song L Feng X. Grand parenting: an analysis framework from grandparents' perspective. J Shaanxi Normal Univ (Philos and Soc Sci Edition). (2018) 47:83–9. 10.15983/j.cnki.sxss.2018.0123

[44] Wu F. Intergenerational conflict and integration: analysis and policy thinking on the differences of ageism groups. Soc Sci in Guangdong. (2013) 5:218–26. 10.3969/j.issn.1000-114X.2013.05.028

